# Weltweit zunehmende Masernfälle sind auch für Deutschland relevant: Das Zentrum für Internationalen Gesundheitsschutz hilft, die globale Gesundheit zu stärken

**DOI:** 10.1007/s00103-020-03248-y

**Published:** 2020-11-24

**Authors:** Sandra Beermann, Luisa Denkel, Johanna Hanefeld

**Affiliations:** 1 Zentrum für Internationalen Gesundheitsschutz, Robert Koch-Institut, Nordufer 20, 10315 Berlin, Deutschland; 2 Abteilung für Infektionsepidemiologie, FG33 – Fachgebiet für Impfprävention, Robert Koch-Institut, Seestraße 10, 10315 Berlin, Deutschland

Die heutige hochmobile und vernetzte Welt bietet unzählige Möglichkeiten für die rasche Ausbreitung von Infektionskrankheiten, wie die aktuelle COVID-19-Pandemie zeigt. Ein Ausbruch oder eine Epidemie in einem Teil der Welt kann nur wenige Stunden davon entfernt sein, an einem anderen Ort zu einer unmittelbaren Bedrohung zu werden. Seit den 1970er-Jahren werden neu auftretende Infektionskrankheiten mit einer beispiellosen Rate von einer oder mehreren pro Jahr identifiziert. Heute gibt es fast 40 Krankheiten, die vor einer Generation noch unbekannt waren.

Darüber hinaus hat die Weltgesundheitsorganisation (WHO) in den letzten 5 Jahren weltweit mehr als 1100 epidemische Ereignisse verifiziert. Die Infektionserreger können jede Grenze überwinden. Epidemien bedrohen die Gesundheit der Bevölkerung, erzeugen großes menschliches Leid und bergen ein enormes wirtschaftliches Schadenspotenzial. Ihre Folgen tragen besonders in ressourcenarmen Ländern zur sozialen und politischen Instabilität bei und können Fluchtbewegungen verstärken. Ebenso führen soziale und politische Instabilität zu Umständen, die das Auftreten neuer Erreger sowie die Verbreitung von Infektionskrankheiten fördern.

Seit der Ebolakrise 2014/2015 in Westafrika, und spätestens durch die aktuelle COVID-19-Pandemie, ist deutlich geworden, wie eng der nationale mit dem internationalen Gesundheitsschutz zusammenhängt. Eine internationale Perspektive ist daher ein wesentlicher Bestandteil einer nationalen Gesundheitspolitik.

Dies spiegelt sich auch in den Erklärungen der Bundesregierung anlässlich des G‑7-Gipfels 2015 in Elmau sowie des G‑20-Gipfels in Hamburg 2017 wider. Deutschland übernimmt darin künftig eine strategische und gestaltende Rolle innerhalb der Staatengemeinschaft zur langfristigen Stärkung von Gesundheit im internationalen Kontext. Für die Umsetzung dieses Ziels hat das Bundesministerium für Gesundheit die Einrichtung eines „Zentrums für Internationalen Gesundheitsschutz – ZIG“ am Robert Koch-Institut (RKI) veranlasst. „Ein leistungsfähiges Gesundheitssystem ist weltweit eine wichtige Grundlage für Stabilität und Mobilität. Durch die Einrichtung eines neuen Zentrums für Internationalen Gesundheitsschutz im RKI stärken wir die Gesundheit der Bevölkerung international und auch in Deutschland“, betonte Bundesgesundheitsminister Jens Spahn zum Start des Zentrums Anfang 2019.

Zu den Hauptaufgaben des ZIG gehört es, den Gesundheitsschutz international nachhaltig zu stärken. Dies erfolgt durch Partnerschaften mit nationalen Public-Health-Instituten und Forschungseinrichtungen sowie mit multilateralen Organisationen, wie der WHO. Wichtige Arbeitsbereiche sind das Informationsmanagement, die Entwicklung evidenzbasierter Methoden sowie die Unterstützung hinsichtlich der Bereitstellung von Laborkapazitäten und der Umsetzung von Projekten zum internationalen Gesundheitsschutz. Das ZIG trägt damit zur Erfüllung der neuen Anforderungen im Bereich des internationalen Gesundheitsschutzes bei, wie sie in der Neufassung des Infektionsschutzgesetzes aus dem Jahr 2017 formuliert sind.

Seit Beginn des COVID-19-Ausbruchs Anfang 2020 unterstützt das RKI auf nationaler und internationaler Ebene die Reaktionen auf die Krise. Beim Erfahrungsaustausch zum Management des COVID-19-Ausbruchs ist das RKI ein gefragter Partner für Public-Health-Institutionen weltweit. So hat es in den letzten Monaten viel Unterstützungsarbeit im Bereich der Diagnostik geleitstet. Das Public-Health-Intelligence-Team des ZIG analysiert kontinuierlich die internationale epidemiologische Situation und steht in regelmäßigem Kontakt mit Gesundheitsministerien und öffentlichen Gesundheitseinrichtungen in zahlreichen Ländern. Durch die Einrichtung des ZIG konnte das RKI mit Stand Anfang September 2020 bereits in über 60 Ländern in Afrika, Asien, Europa und Lateinamerika Unterstützung und Beratung zu allen Aspekten der öffentlichen Gesundheit bei der Reaktion auf Ausbrüche leisten.

Doch nicht nur neue Infektionserreger, sondern auch altbekannte spielen in vielen Ländern noch immer eine große Rolle. Vor 50 Jahren glaubten Fachleute, Infektionskrankheiten endgültig besiegen zu können. Mit der Entwicklung von Impfstoffen und Antibiotika hat die Wissenschaft große Fortschritte im Kampf gegen Infektionskrankheiten gemacht. Impfungen bieten einen effektiven Schutz vor Maserninfektionen und deren möglichen Komplikationen. Masern gehören weltweit zu den Infektionen mit der höchsten Sterblichkeit im frühen Kindesalter. Daher hatte sich die WHO im Jahr 2010 zum Ziel gesetzt, die Erkrankung bis 2015 in Europa auszurotten. Um dieses Ziel zu erreichen, müsste bei 95 % der Bevölkerung eine ausreichende Immunität vorliegen [1]. Bis heute stagniert jedoch die globale Impfquote der 1. Masernimpfdosis bei 86 % und keine der 6 WHO-Regionen konnte die Masernelimination bisher erreichen oder aufrechterhalten [2]. Obwohl seit vielen Jahren ein sicherer und effektiver Impfstoff verfügbar ist, nehmen die Masernfälle seit 2018 sogar weltweit wieder zu.

Die Gründe für den erneuten globalen Anstieg der Masernfallzahlen sind vielfältig. In dieser Ausgabe des Bundesgesundheitsblattes führt der Artikel „Die globale Masernkrise – Ursachenvielfalt von bewaffneten Konflikten bis Impfskepsis“ mögliche Gründe beispielhaft auf. Es wird auf strukturelle und psychologische Barrieren der Masernbekämpfung eingegangen. Auch hier zeigt sich die Verknüpfung zwischen strukturellen Gegebenheiten und globaler Gesundheit.

Strukturelle Barrieren, wie zum Beispiel die Unterbrechung von Routineimpfprogrammen durch politische Unsicherheiten, sind vor allem in fragilen Ländern wie Madagaskar, der DR Kongo und der Republik Jemen die Hauptursache für geringe Masernimpfquoten und hohe Masernfallzahlen in der gesamten Bevölkerung. Der Zugang zu medizinischen Dienstleistungen, einschließlich Impfangeboten, kann durch humanitäre Krisen und Instabilität kurz- oder langfristig in Ländern oder Landesteilen für die gesamte Bevölkerung oder Subpopulationen erschwert bzw. verhindert sein. Auch können strukturelle Barrieren für bestimmte Subpopulationen bestehen. So sind indigene Populationen in *Venezuela* und *Brasilien* sehr stark von Masernausbrüchen betroffen. Humanitäre Hilfe, einschließlich Impfungen, ist in dieser vulnerablen Population aufgrund der Abgeschiedenheit der Siedlungsgebiete und der seminomadischen Lebensweise nur begrenzt möglich.

Psychologische Barrieren wie mangelndes Vertrauen und fehlende Risikowahrnehmung sind in vielen Ländern eine wichtige Ursache für den Anstieg der Masernfallzahlen. Dies betrifft insbesondere Länder mit sehr gut funktionierenden Gesundheitssystemen und einem hohen Lebensstandard, ist aber auch in Ländern mit einem mittleren oder niedrigen Pro-Kopf-Einkommen (z. B. Philippinen, DR Kongo) zu beobachten.

Der Artikel zieht als Fazit, dass die kontinuierliche Bewertung der geschilderten Faktoren der Masernbekämpfung sowie die Entwicklung und Umsetzung weiterer geeigneter Maßnahmen essenziell sind, um das Ziel der weltweiten Eradikation der Masern erreichen zu können. Denn ansonsten bleibt auch in Ländern mit einer verifizierten Elimination der Masern das Risiko permanent bestehen, Masernfälle zu importieren, die zu Ausbrüchen führen können.
